# Enhanced thermal conductivity of epoxy composites filled with silicon carbide nanowires

**DOI:** 10.1038/s41598-017-02929-0

**Published:** 2017-06-01

**Authors:** Dianyu Shen, Zhaolin Zhan, Zhiduo Liu, Yong Cao, Li Zhou, Yuanli Liu, Wen Dai, Kazuhito Nishimura, Chaoyang Li, Cheng-Te Lin, Nan Jiang, Jinhong Yu

**Affiliations:** 1 Faculty of Materials Science and Engineering, Kunming University of Science and Technology, Kunming, 650093 China; 2 Key Laboratory of Marine Materials and Related Technologies, Zhejiang Key Laboratory of Marine Materials and Protective Technologies, Ningbo Institute of Materials Technology and Engineering, Chinese Academy of Sciences, Ningbo, 315201 China; 3 College of Materials Science and Engineering, Guilin University of Technology, Guilin, 541004 China; 4 Advanced Nano-processing Engineering Lab, Mechanical Systems Engineering, Kogakuin University, Kochi, 780-0805 Japan; 5 Research Institute & School of Systems Engineering, Kochi University of Technology, Kami City, Kochi 782-8502 Japan

## Abstract

In this study, we report a facile approach to fabricate epoxy composite incorporated with silicon carbide nanowires (SiC NWs). The thermal conductivity of epoxy/SiC NWs composites was thoroughly investigated. The thermal conductivity of epoxy/SiC NWs composites with 3.0 wt% filler reached 0.449 Wm^−1^ K^−1^, approximately a 106% enhancement as compared to neat epoxy. In contrast, the same mass fraction of silicon carbide micron particles (SiC MPs) incorporated into epoxy matrix showed less improvement on thermal conduction properties. This is attributed to the formation of effective heat conduction pathways among SiC NWs as well as a strong interaction between the nanowires and epoxy matrix. In addition, the thermal properties of epoxy/SiC NWs composites were also improved. These results demonstrate that we developed a novel approach to enhance the thermal conductivity of the polymer composites which meet the requirement for the rapid development of the electronic devices.

## Introduction

The rapidly-developing electronic industry focuses on at miniaturizing and lightening of electronic equipment. Meanwhile, performance and versatility of electronic devices have been integrated with a decreasing dimensions. Consequently, a high level of circuit integration cause high heat, which needs to be rapidly dissipated to let the device work efficiently^1^. Epoxy resins are widely applied in the aerospace, automotive and electronic industries for supporting and adhesive, owing to their excellent mechanical properties, thermal stability, and chemical resistances^2, 3^. However, intrinsic low thermal conductivity of neat epoxy resins (only the order of 0.10 Wm^−1^ K^−1^ at room temperature)^4^ limits their applications^5^. In order to overcome this problem, a variety of methods have been developed to improve the thermal conductivity. A traditional method is to introduce high contents of thermally conductive fillers, such as metals^6–11^, ceramic particles^12–15^, carbon nanotubes^16–20^, or graphene nanoplateles^21–25^. In particular, the silicon carbides are provided with excellent properties such as high thermal conductivity, high thermal stability, high breakdown field, excellent mechanical properties and chemical inertness^26–32^. Therefore, they can be applied in various circumstances such as in high temperatures, high electron density, high frequency and in harsh environments^33^. The high aspect ratio of silicon carbide nanowires (SiC NWs) makes it favorable to be used as filler in epoxy resin, in order to achieve the desired properties.

Efficiently cooling of electronic devices requires minimal coefficient of thermal expansion (CTE) mismatch of the heat source and heat sink. Nowadays, the heat source are usually Si-based^1^. Copper and aluminum are traditional heat dissipating materials, which show large mismatch with silicon and insulating ceramics and cannot directly attach to silicon without stress compensating interlayers^34^. Therefore, control of reinforcing fillers into the polymer can fulfill the high thermal conductivity and the lowest CTE or thermal distortion parameter (TDP, an indicator of thermal dilation) potential requirements. It will meet the requirement of thermal management and electronic packaging applications ranging from microelectronic components and devices to supercomputers^1, 35, 36^.

It is noted that so far only few works have been reported about the application of epoxy/SiC NWs composites^37–47^. Therefore, detailed investigation of epoxy/SiC NWs for thermal management or heat dissipation is needed, which is still in its infancy. Herein, we proposed a rapid and simple strategy for the preparation of epoxy/SiC NWs composites. The SiC NWs were mixed uniformly with epoxy using ethanol as the solvent. Subsequently, the mixture was stirred in water bath and curing agent was added to obtain the epoxy/SiC NWs composites. Based on this method, the SiC NWs loading can be easily controlled, and the interconnected network of nanowires in the composites results in not only enhanced thermal properties but also retained low CTE performance.

## Materials

Silicon carbide nanowires were produced by Changsha Sinet Advanced Materials Co., Ltd. China. Silicon carbide micron particles with the particle size about 1 μm were purchased from Shanghai St-Nano Science and Technology Co., Ltd. China. Anhydrous ethanol was produced by Sinopharm Chemical reagent Co., Ltd. Cycloaliphatic epoxy resin (6105, DOW Chemicals) along with hardener of methyl-hexahydrophthalic anhydride (MHHPA, Shanghai Liyi Science & Technology Development, China) was used in the present study. Neodymium (III) acetylacetonatetrihydrate (Nd(III)acac) purchased from Aldrich Chemicals was used as latent catalyst.

### Preparation of epoxy composites

**Figure 1:**
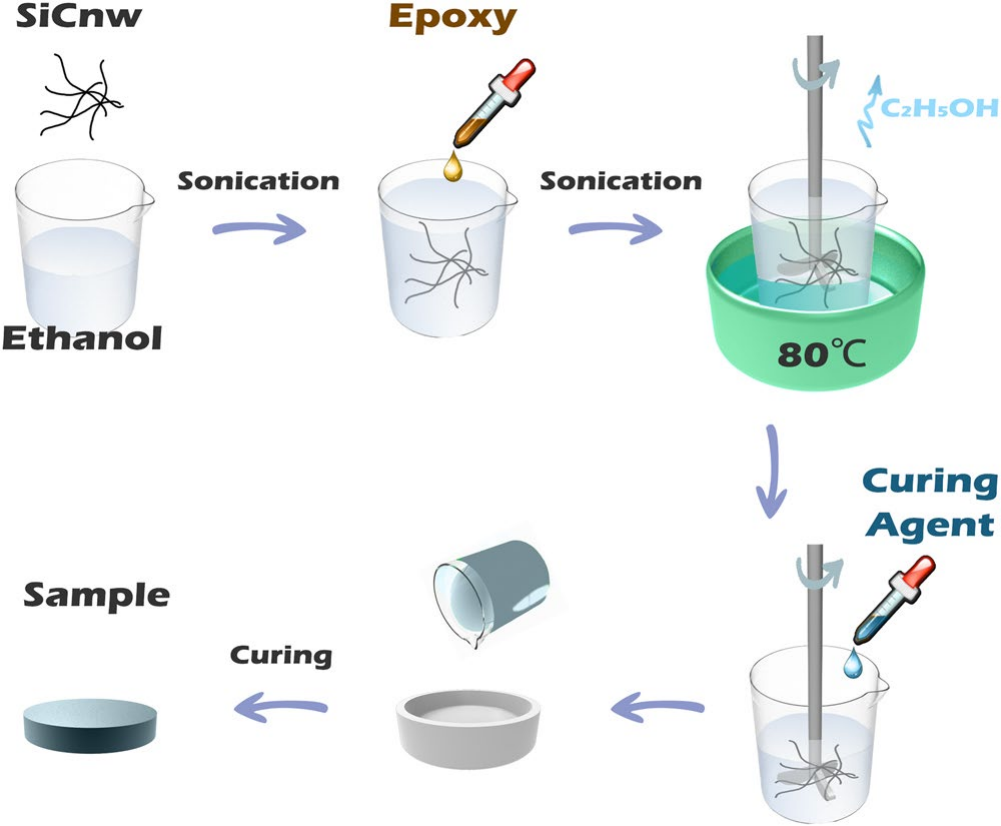
The preparation process of epoxy/SiC NWs composites.

Epoxy composites with different SiC NWs loadings were prepared by the following procedures. Required quantity of Nd(III)acac was added into a cycloaliphatic epoxy resin and subsequently stirred at 80 °C in a three-necked flask for 2 h. The homogeneous solution was then cooled down to ambient temperature. A desired amount (0.5, 1.0, 1.5, 2.0, 2.5, and 3.0 wt%) of SiC NWs was dispersed in ethanol in an ultrasonic bath for 0.5 h and then added into the predetermined amount of epoxy resin. The obtained mixture was then placed in a beaker with vigorous mechanical stirring at 80 °C in water bath until complete evaporation of ethanol. Curing agent was added at a ratio of 100:95 (epoxy:curing agent) by weight into the beaker and was stirred for 20 min. It was further degassed in the vacuum oven for 10 min to remove air bubbles. Finally, the mixture of epoxy with the homogeneously dispersed SiC NWs was poured into the mold to cure at 135 °C for 2 h and 165 °C for 14 h. After the curing process, the samples were naturally cooled down to the room temperature and then polished with emery paper for different characterizations. For comparison, the epoxy composites with SiC MPs were also fabricated with the same procedures as described above. For the sake of convenience, the composites containing SiC NWs and SiC MPs were denoted as epoxy/SiC NWs composites and epoxy/SiC MPs composites, respectively. The preparation process of epoxy/SiC NWs composites is illustrated in Fig. 1.

### Characterizations

Atomic force microscope (AFM) measurement was conducted on a multimode SPM from digital instruments with Nanoscope IA controller. X-ray photoelectron spectroscopy (XPS) was carried out with a Kratos AXIS Ultra DLD spectrometer, using AIKα excitation radiation (hγ:1253.6 eV). The X-ray diffraction (XRD) patterns of the samples were recorded on a D8 DISCOVER with GADDS (BRUKER Ltd. Germany) with CuKα radiation (λ = 1.5406 Å). The scanning was performed from 10° to 80° with a speed of 4° min^−1^ at room temperature. The fractured surface of the composites was examined by field emission scanning electron microscopy (FE-SEM, QUANTA FEG250, USA) at an acceleration voltage of 20 kV. Samples that were broken and the fractured surface was coated with a thin gold layer to avoid accumulation of charge. The microstructures of the samples were obtained from JEOL JEM-2100 (TEM, JEOL, Japan) instrument with an acceleration voltage of 200 kV. Thermal conductivities of the composites were measured using the light flash apparatus LFA 447 NanoFlash® (NETZSCH, Germany). The IR-photos were captured by infrared camera (Fluke, Ti400, USA). Differential scanning calorimetry was performed by a Pyris Diamond DSC (Perkin-Elmer, USA) from 20 to 250 °C at a heating rate of 10 °C/min under a nitrogen atmosphere to study glass transition temperature (Tg) of the composites. The CTE measurements were performed on a thermal mechanical analyzer (TMA 402F1/F3, NETZSCH, Germany) from 310 to 460 K at a heating rate of 5 K/min. Thermogravimetric analysis (TGA) was carried out using TG 209 F3 thermo-analyzer (NETZSCH, Germany). The temperature range was from 50 to 800 °C at a ramp rate of 10 °C/min in nitrogen atmosphere.

## Results and Discussion

### Characterization of SiC nanowires and micron particles

**Figure 2:**
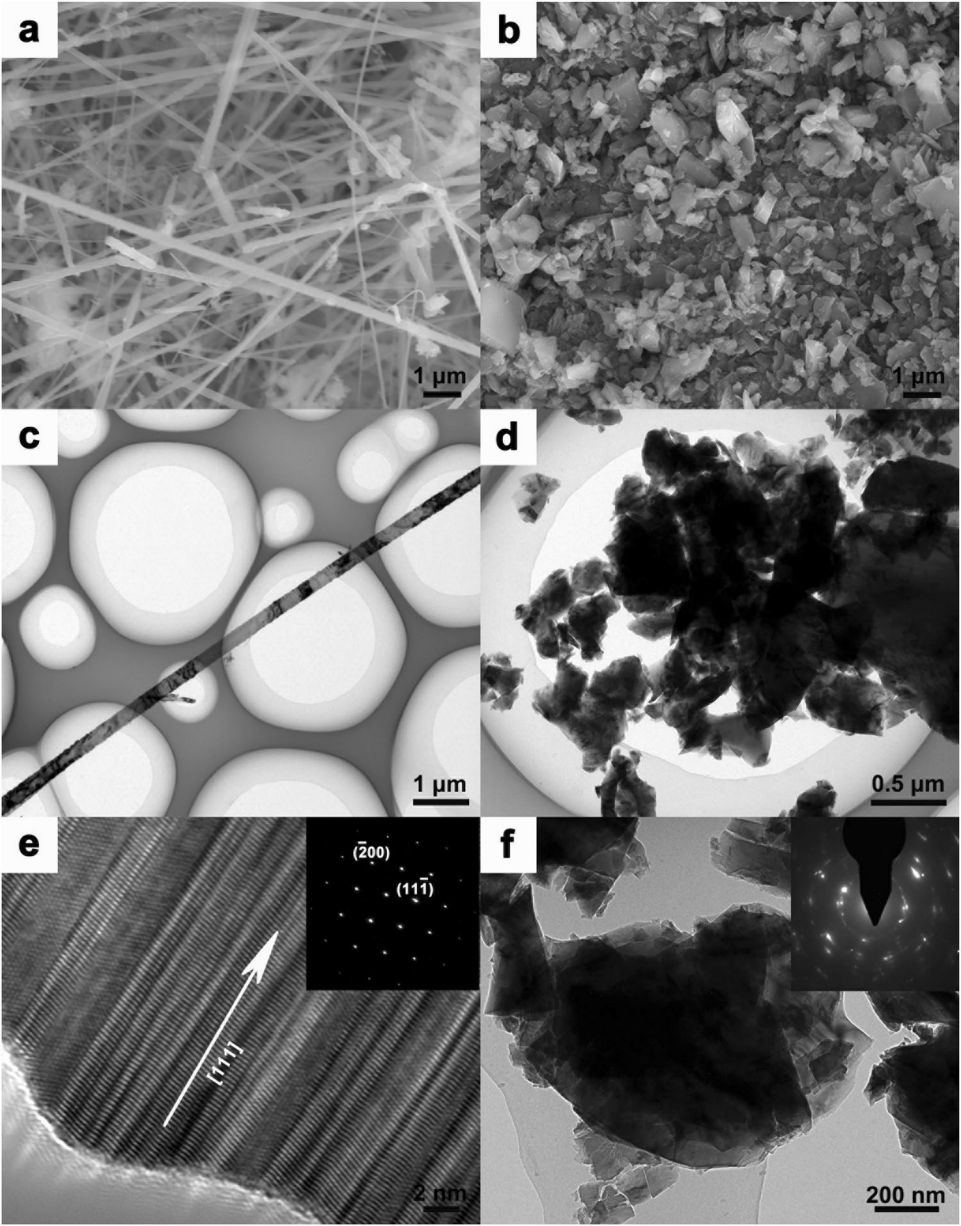
SEM images of (**a**) SiC NWs and (**b**) SiC MPs; TEM images of (**c**) SiC NWs and (**d**) SiC MPs; HR-TEM images of (**e**) SiC NWs and (**f**) SiC MPs. The SAED pattern images of SiC NWs and SiC MPs are show in the inset of (**e**) and (**f**).

Figure 2a shows the typical SEM image of the SiC NWs. The SiC NWs are mainly straight and intertwined nanowires with the diameter range from 150 to 250 nm. The average length of nanowires is difficult to determine, but it can be estimated that substantial amounts exceed a length of 120 μm. The aspect ratio is approximately from 480 to 800 (see Supplementary Figure S1a). Meanwhile, large scale SEM images and SiC suspension photo are shown in Supplementary Figure S1. The TEM image (Fig. 2c) shows an individual long and straight SiC NWs with smooth surface and very homogeneous diameter of SiC NWs on the copper grid, and fairly clean with very few particles attached to its surface, which is in agreement with the observation in SEM. The HR-TEM image of the nanowire tip displays the spacing distance between each parallel stripe (d = 0.25 nm) equal to interplanar spacing of SiC cubic crystal structures, and the nanowire grow along the direction of [111] which is perpendicular to the parallel stripes. Along the axis of the SiC NWs, the high density of stack-faults and twin crystal structure with about a few nanometers wide can be observed. The corresponding SAED pattern with the zone axis index of [011] can be indexed with the indices ($\overline{2}00$) and ($11\overline{1}$) of crystal faces. This indicates a unit face centered cubic lattice β-SiC structure (lattice parameter = 4.38 Å) and is consistent with the diffraction pattern. The SiC NWs are β-SiC forms based on HR-TEM and SAED analysis, as shown in Fig. 2e. In contrast to SiC NWs, the SEM image (Fig. 2b) of SiC MPs reveals an irregular shape with abundant corner angles and the vast majority of SiC MPs with the average size is less than 1 μm. A representative TEM image (Fig. 2d) of the SiC MPs shows that the particles are attached together with a uniform size ranging from 0.1 to 2 μm. Figure 2f displays the HR-TEM image and a corresponding SAED pattern of the SiC MPs. It is hardly to observe clearly crystal lattice in the HR-TEM image, which indicates polycrystalline structure of SiC MPs.

**Figure 3:**
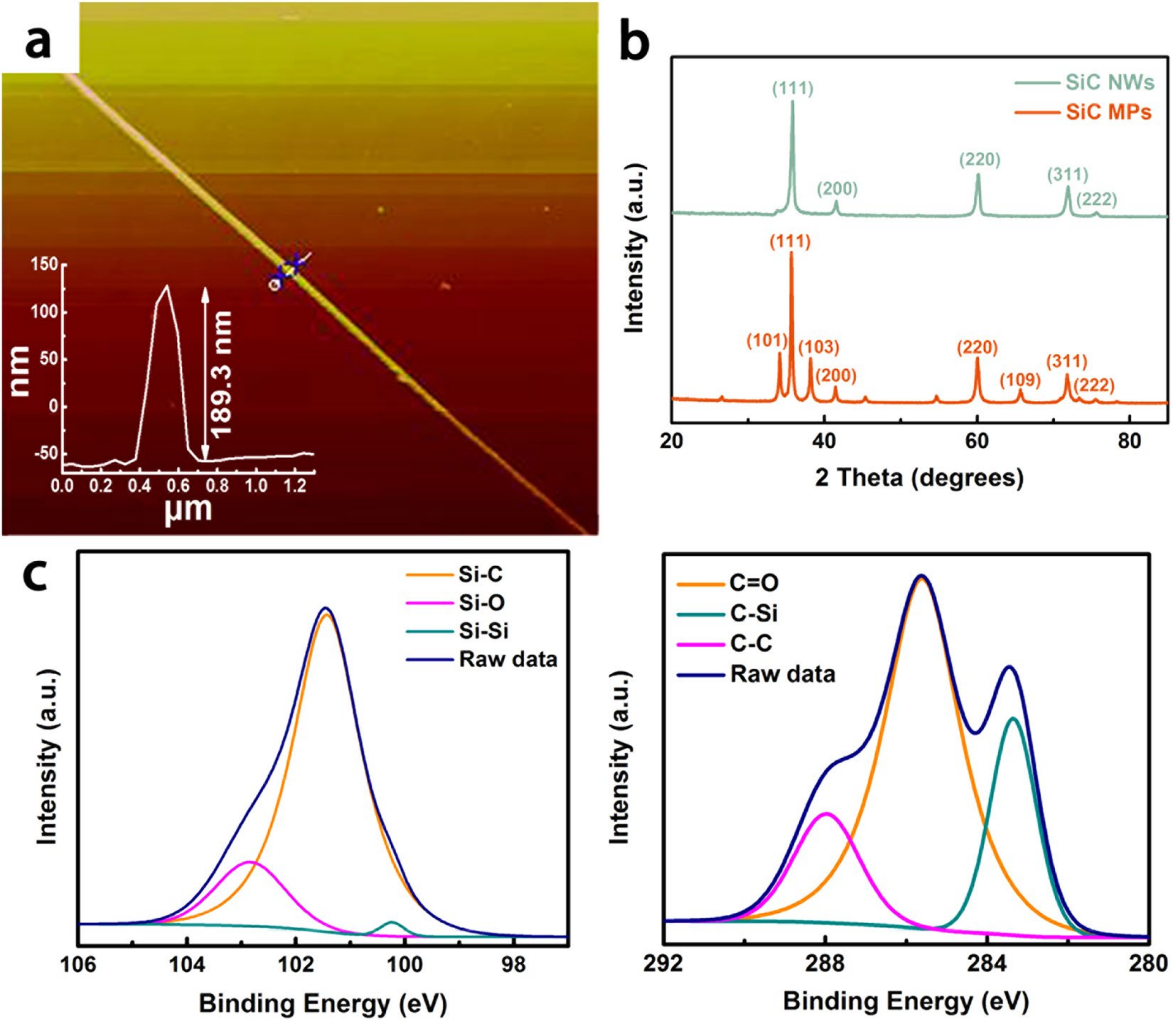
(**a**) AFM image; (**b**) XRD pattern; XPS spectra of SiC NWs: (**c**) Si_2p_ and (**d**) C_1s_.

Figure 3a shows an AFM image of single SiC NWs with a diameter of 189.3 nm. This result is also supported by previous analysis of SEM and TEM. XRD patterns of SiC NWs and micron particles are shown in Fig. 3b. In the SiC NWs pattern, five peaks can be indexed as the (111), (200), (220), (311) and (222) reflections of the face-centered cubic cell of β-SiC at 2θ = 35.8°, 41.6°, 60.2°, 71.9°, and 75.6°. Therefore, the phase of SiC NWs classified is 3C-SiC (β-SiC) with the lattice parameter α = 4.35 Å, which is matched well with the reference data (JCPDS card No. 73–1708). By combining with the above results and XRD analysis of the SiC NWs, it can be concluded that the nanowires are composed of single-crystalline β-SiC^48^. However the diffraction pattern of SiC MPs is complicated. Except for the above the five peaks resulting from β-SiC, several other peaks can be observed, indexing as the (101), (103) and (109) reflections of hexagonal polytypes 6H-SiC (α-SiC), lattice parameters of 6H-SiC calculated by least squares method from the positions of the peaks observed, as follows: α = 3.078 Å, c = 15.12 Å. We speculated that SiC MPs are polycrystalline, which is in good agreement with the previous conclusion^49^. XPS analysis is employed to further verify the surface composition and investigate the functional groups and the Si_2p_ and C_1s_ high-resolution results are shown in Fig. 3c and d. For the case of Si_2p_, the spectrum can be decomposed to three Gaussian components located 100.2, 101.4 and 102.8 eV, originated from Si-Si, Si-C, and Si-O, respectively. The spectra of C_1s_ compose of three bonds centered at 283.3, 285.6, and 287.9 eV, which corresponds to C-Si, C-C, and C=O, respectively. A small amount of C=O can be attributed to the adsorbed CO_2_ on the SiC NWs surface. According to XPS quantification analysis, Si and C atomic ratio of β-SiC nanowire is approximately 1.0:1.2^50, 51^.

### Microstructure of composites

**Figure 4:**
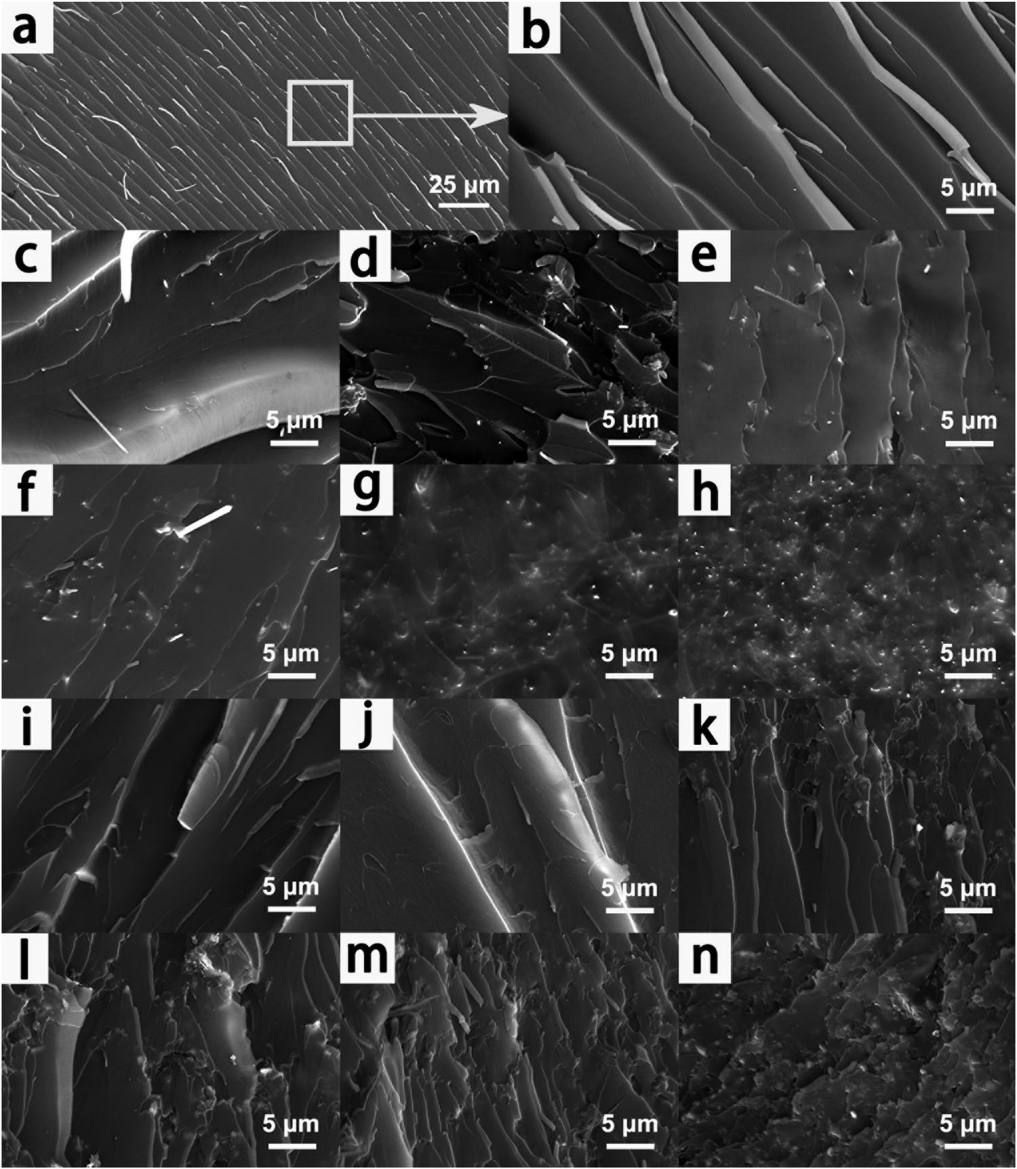
SEM images of neat epoxy: (**a**) and (**b**); epoxy/SiC NWs: (**c**) 0.5 wt%, (**d**) 1.0 wt%, (**e**) 1.5 wt%, (**f**) 2.0 wt%, (**g**) 2.5 wt% and (**h**) 3.0 wt%; epoxy/SiC MPs composites: (**i**) 0.5 wt%, (**j**) 1.0 wt%, (**k**) 1.5 wt%, (**l**) 2.0 wt%, (**m**) 2.5 wt%, and (**n**) 3.0 wt%.

To understand the relationship between the structure and properties of epoxy composites, the fracture surfaces of neat epoxy and epoxy composites are characterized by SEM, as illustrated in Fig. 4. Figure 4(a,b) depict striped structures with cracks, which displays the river like patterns, and similar patterns can be also found on the fracture surface of neat epoxy shown in Fig. 4(c–f). In addition, the regions of the fracture surface are very smooth, reveals that the composite is a brittle thermosetting polymer. It can be clearly observed that the fracture surfaces of the epoxy composites exhibit considerably different fracture graphic features with the increasing incorporation of the SiC NWs. Figure 4(g,h) show that the pattern of cross section is very smooth with the homogeneously dispersion of SiC NWs and almost no obvious naked nanowires. It is suggested that the nanowires formed a crosslinked network and a strong interfacial interaction between epoxy matrix and surface of nanowires, which can be concluded as the critical factor for thermal properties. By comparison, the epoxy/SiC MPs composite show river patterns appearing on the fracture surface close to neat epoxy. Different from epoxy/SiC NWs composites, there are some cracks on the regions of the fracture surface in epoxy/SiC MPs composites as shown in Fig. 4(i,j). Meanwhile, with the increasing amounts of SiC MPs in epoxy, a rougher fracture surface and numerous tortuous indentations and deep cracks can be observed, shown in Fig. 4(k–n). Unlike epoxy filled with SiC NWs, the SiC MPs protrude cleanly from the fracture surface, indicating a week interfacial interaction between epoxy matrix and SiC MPs.

### Thermal properties of neat epoxy and its composites

**Figure 5:**
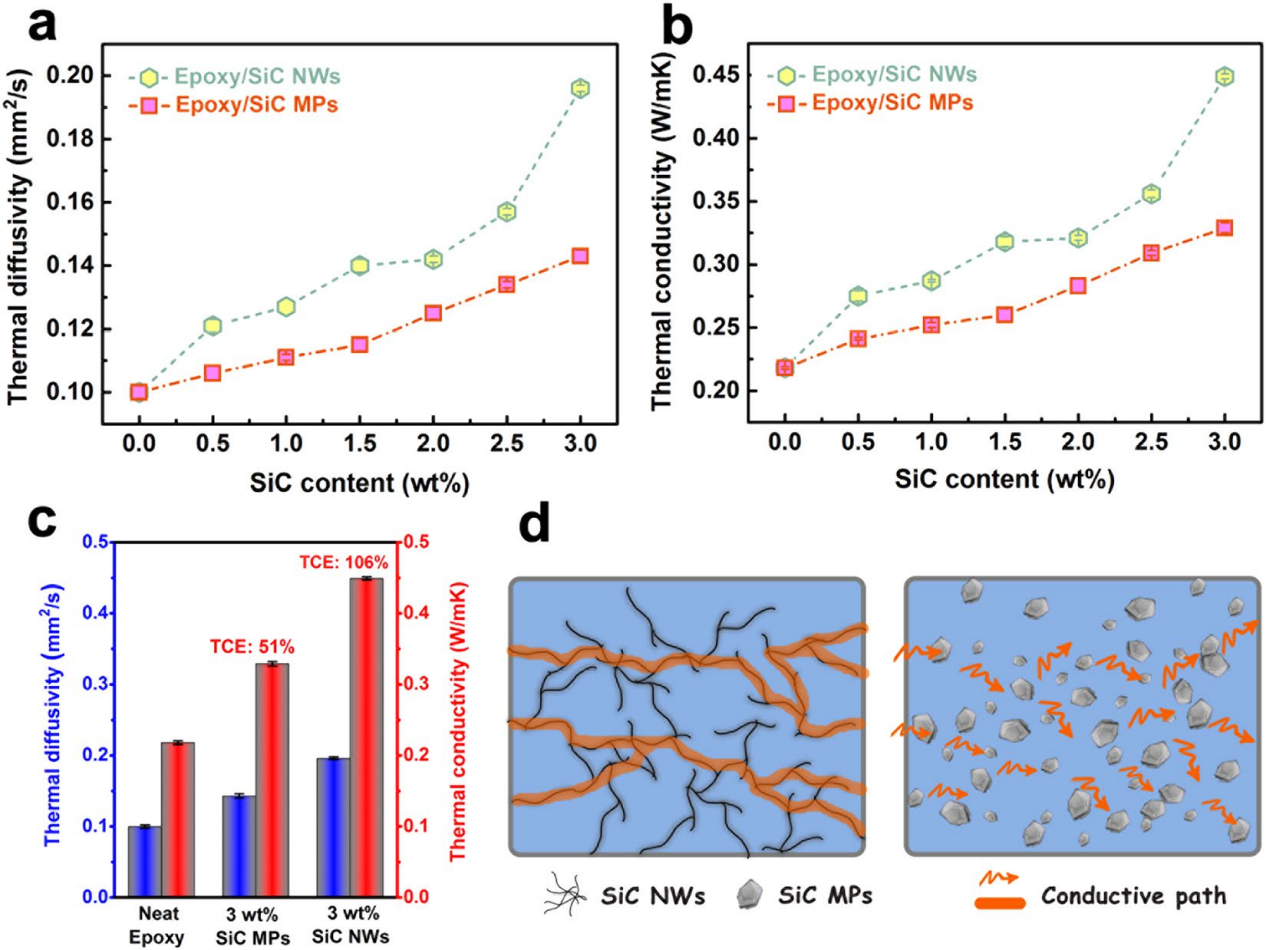
(**a**) Thermal diffusivity and (**b**) thermal conductivity as a function of SiC NWs or SiC MPs content; (**c**) Thermal conductivity enhancement (TCE) of epoxy composites with 3 wt% filler compared to neat epoxy; (**d**) The model of heat flow for the epoxy composites.

Recently, it has been reported that the fillers with high aspect ratio, such as nanowires and nanosheets, can form more continuous thermally conductive network in the polymer matrix and therefore are more efficiently in improving the heat transfer^1, 52^. In order to fabricate composites with superior thermal conductivity, beside the amount of filler, uniform dispersion in polymer matrix is the critical factor^35^. We adopted a transient laser flash method to indirectly estimate the thermal conductivity of the neat epoxy and its composites at room temperature. In order to assess the effect of SiC fillers on the thermal properties of composites, thermal diffusivity was measured first and then thermal conductivity was calculated as a function of SiC fillers loading content (0–3 wt%). The dependence of thermal diffusivity and thermal conductivity on SiC loading is presented in Fig. 5a and b respectively. In Fig. 5a and b both the thermal diffusivity and conductivity of the samples shows monotonically increase as more SiC were incorporated. As can be observed, the thermal diffusivity of pristine epoxy is around 0.108 mm^2^/s. With the addition of 0.5 wt% SiC NWs, the thermal diffusivity of epoxy/SiC NWs composite increased to 0.121 mm^2^/s with about 11% enhancement. As the content of SiC NWs is further increased to 3 wt%, the thermal diffusivity is improved from 0.108 mm^2^/s to 0.196 mm^2^/s, and the thermal conductivity was improved from 0.218 Wm^−1^ K^−1^ to 0.449 Wm^−1^ K^−1^. At 3 wt% SiC NWs loading, the thermal conductivity of epoxy/SiC NWs composite is improved significantly by 106% compared to that of neat epoxy, as illustrated in Fig. 5c. Moreover, a sharp increase on thermal diffusivity and thermal conductivity is observed when the loading fraction increased from 2.5 to 3.0 wt%. It is speculated that the percolation threshold is obtained at ~2.5 wt% and continuous SiC NWs network is formed. For comparison, the thermal conductivity of epoxy/SiC MPs composites were also determined with the same procedure. The value is 0.329 Wm^−1^ K^−1^ at 3.0 wt%, lower than that of epoxy/SiC NWs composites at the same loading fraction, only enhance 51% comparing to that neat epoxy, as shown in Fig. 5b and c. It could be main reasons as following: (a) the well-bridged and efficient thermal conduction network between nanowires and nanowires; (b) SiC NWs with larger aspect ratio than SiC MPs and (c) a better interaction between SiC NWs with the epoxy matrix^27^.

It is well known that the phonons play a major role in heat conduction of majority solid materials. Meanwhile, both the harmonic or anharmonic phonon-phonon interaction at high temperatures and the scattering of the phonons by the crystal boundaries at low temperatures determine the thermal conductivity of semiconductors^53^. Neat epoxy cannot obtain an expected thermal conductivity because the low crystallinity and phonon scattering of the randomly entangled moleculechains^54^. Introducing high thermal conductivity SiC NWs (~100 Wm^−1^ K^−1^)^55^ into epoxy is an effective approach to enhance the thermal conductivity of the composites. In general, the low loading SiC NWs in the polymer matrix are in an isolated state which is analogous to the “sea-island” structure. If the quantity of SiC NWs is further increased and reaches the percolation threshold, local chain and network would mutually bridge to generate the whole network. The formation of efficient network that contributes epoxy and SiC NWs to fabricate continuous phases, resulting in enhancing the thermal conductivity of composites^56^. In order to better demonstrate the superiority of SiC NWs as compared with SiC MPs, the thermal conduction visualized model of epoxy composites was proposed, as shown in Fig. 5d. Meanwhile, It can also explain that the SiC MPs are separated by the polymer layer which hinder the SiC MPs forming efficient thermal conduction network in epoxy, even when the loading is 3.0 wt%. It leads to low thermal conductivity values of epoxy/SiC MPs composites in comparison with epoxy/SiC NWs composites. Moreover, the phonon mismatch between SiC MPs and epoxy matrix leads to a large thermal interface resistance. Phonon mismatch indicates that phonon can hardly absorbed by crystalline structure in the procedure of transmission, which means, the energy phonon carries is not sensitively to the crystal lattice, which is in accordance with the aforementioned SEM results (as shown in Fig. 4k,l)^57^.

**Figure 6:**
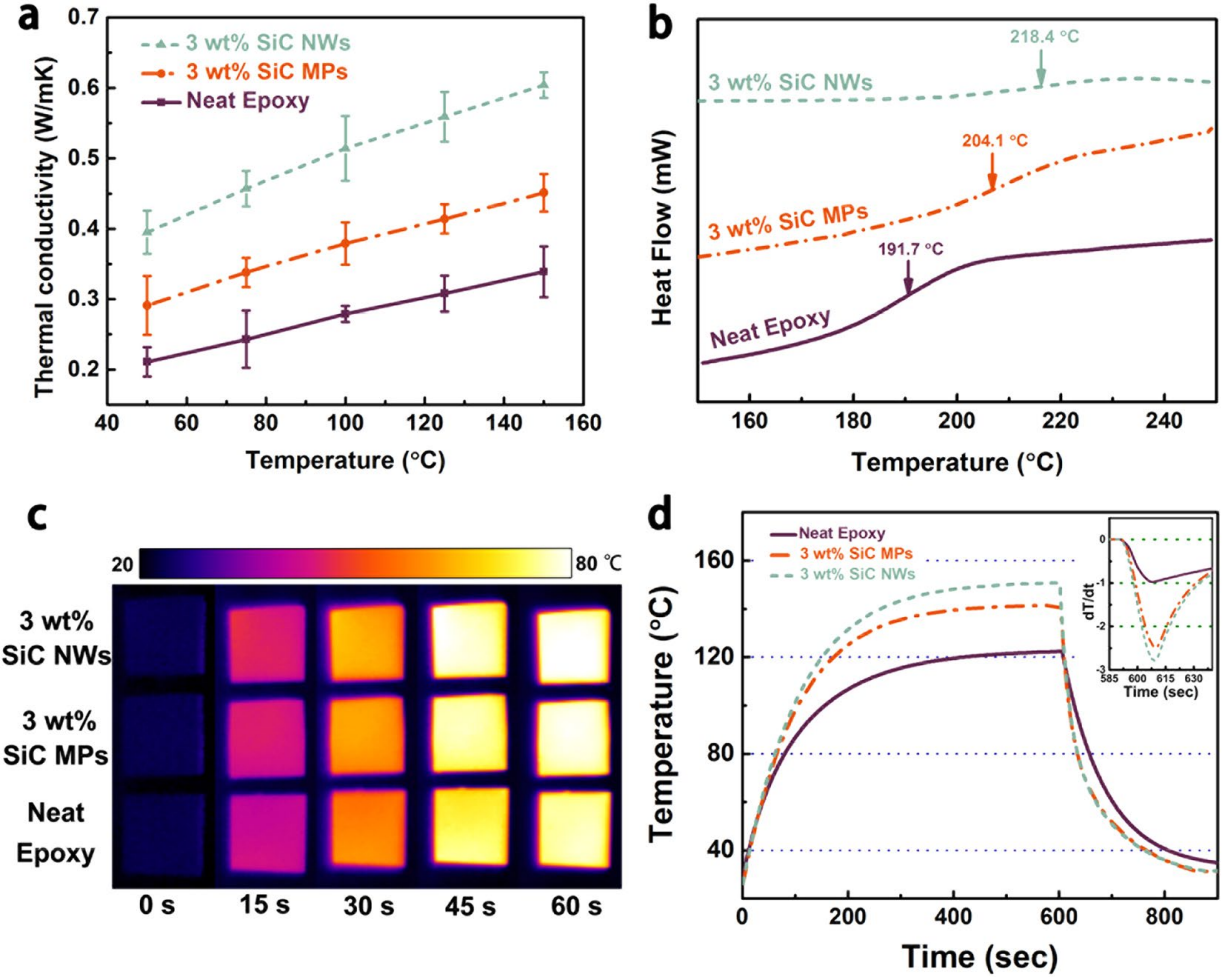
(**a**) Thermal conductivity of neat epoxy and epoxy composites as a function of test temperature; neat epoxy and epoxy composites: (**b**) DSC curves, (**c**) infrared images, (**d**) Surface temperature variation with time upon heating and cooling event.

The effect of temperature on thermal conductivity is investigated at 3 wt% loading of SiC NWs, as shown in Fig. 6a. In the shown pattern, the thermal conductivity of composites is in the order of neat epoxy < epoxy/SiC MPs < epoxy/SiC NWs. For neat epoxy, thermal conductivity is found to be 0.211 Wm^−1^ K^−1^ at 50 °C and increased with temperature over the temperature range investigated. Similarly, the thermal conductivity of epoxy composites shows the same trend with the increase of temperature. Under Tg (shown in Fig. 6b), thermal conductivity is affected by the variation of phonon mean free path caused by structure scattering and chain defect scattering^58, 59^, which is refer to the propagation of lattice wave, independent of temperature, and the defects introduced by blending in the materials system, respectively. When temperature increases, the polymeric chain surrounding the fillers begins to vibrate and straighten out, therefore, the mean free path and phonon propagation length increased. The experimental results show that the longer phonon propagation length implies the higher thermal conductivity. The higher thermal conductivity of epoxy composites at elevated temperature is due to efficient thermal transfer. The result is expected to be very useful as the next generation thermal interface materials^60^.

It is well-known that the Tg of epoxy and its composites is bound up with the cross-linking density^61^. The glass transition temperatures appear over a wide temperature range, because the level of cross-linking in the epoxy matrix is not accordant. Thus onset temperature, intermediate temperature and end temperature can be characteristic temperature for Tg. Herein, the intermediate temperature is defined as Tg. Figure 6b shows the DSC of the neat epoxy and epoxy/SiC NWs at 3 wt% SiC NWs loading, respectively. The glass transition of the samples was investigated in temperature range 25–250 °C. It is observed that the Tg of neat epoxy is 191.7 °C. While the Tg of the epoxy/SiC MPs and epoxy/SiC NWs is 204.1 °C and 218.4 °C, respectively. The Tg of the epoxy/SiC MPs composite is enhanced by 12.4 °C. At the same contents (3 wt%), the Tg of epoxy composite incorporated with SiC NWs enhanced by 26.7 °C. The glass transition temperatures is shifted to higher temperature with the addition of SiC MPs and SiC NWs into the epoxy matrix indicating that a strong interface by reacting with matrix molecules during curing process. This creates more barriers to restrict the motion of macromolecular chain, leading to the higher Tg and promoting thermal stability. The positive effect can also be found from TGA curves of epoxy composites (see Supplementary Figure S2). The results suggest that the SiC NWs or SiC MPs filled in epoxy matrix can restrict thermal motion of the polymer chains and the mobility of the polymer fragment at the interfaces of the epoxy^62^.

In order visually verify the heat transmission property of neat epoxy and epoxy composites, the infrared camera is used to capture the samples surface temperature with increasing heating time, as shown in Fig. 6c. Three samples are vertically placed on the same heater in order of 3 wt% epoxy/SiC MPs, 3 wt% epoxy/SiC NWs and neat epoxy. The surface temperature of the hot plate increased form room temperature to 100 °C. It is observed that the surface of the sample become more and more bright with increasing heating time. Particularly, the color of epoxy composites is quite brighter comparing to neat epoxy. After 60 s, the surface of the epoxy/SiC NWs is brighter, as compare to epoxy/SiC MPs composite. The surface temperature of epoxy/SiC NWs is 79.6 °C and that of epoxy/SiC MPs is 77.4 °C. In contrast, neat epoxy shows a slight orange color and its surface temperature is 74.3 °C. The results give us an interpretation that the better heat dissipation ability of epoxy composites, especially the epoxy/SiC NWs composites, which is in good agreement with the thermal conductivity values shown in Fig. 5b. To further demonstrate the effect on thermal performance, the system with a hot plate, a thermocouple element and versatile voltmeter are employed to get quantitative results of heating and cooling process. As shown in Fig. 6d, it can be noticed that the samples of epoxy/SiC MPs and epoxy/SiC NWs reach their highest temperatures at 141.6 and 150.8 °C. While neat epoxy only rises to 122.3 °C by the end of 602 s, which is consistent with infrared images. After 602 s heating, the samples are shifted to a room temperature plate immediately. The cooling data with differential treatment ranging from 601 to 750 s are shown in the inset of Fig. 6d. Obviously, we found that epoxy/SiC NWs composites is the fastest one cooling down to room temperature, followed by epoxy/SiC MPs, and neat epoxy. In short, epoxy composites show better heat dissipating behaviors comparing with neat epoxy. Among all of composites, epoxy/SiC NWs composites are illuminated to be the most excellent, which indicates that SiC NWs are the most promising filler to enhance the thermal propagation of epoxy composite.

**Figure 7:**
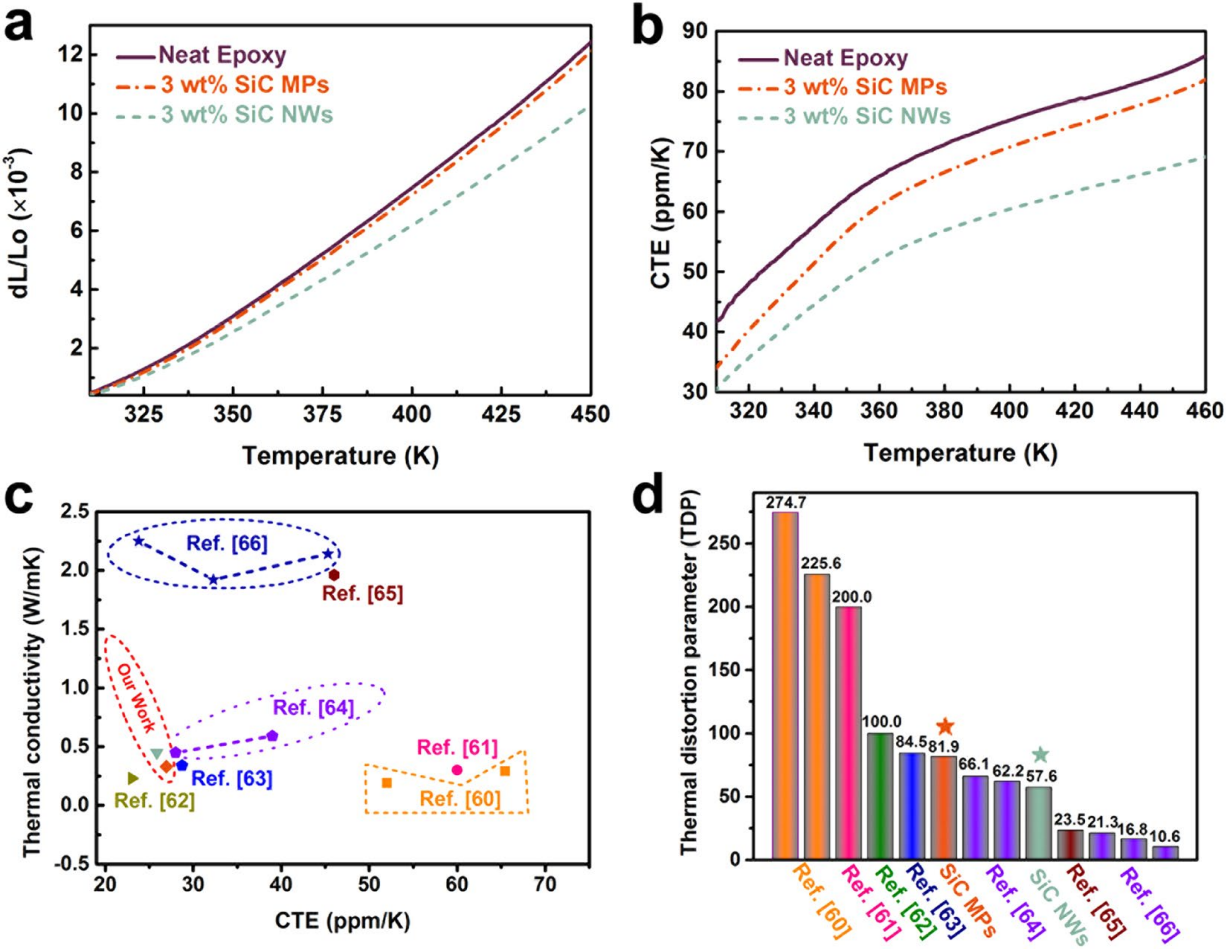
TMA curves of neat epoxy and epoxy composites: (**a**) thermal strain curves, (**b**) CTE curves; Comparison of (**c**) thermal conductivity versus CTE and (**d**) TDP of the epoxy/SiC NWs and epoxy/SiC MPs composites with various engineering materials.

Figure 7a and b show comparative thermal mechanical analysis (TMA) results of neat epoxy and epoxy composites with 3 wt% filler. The epoxy/SiC NWs composite exhibit lower thermal strain comparing to the epoxy/SiC MPs and neat epoxy. It can be noted that the thermal strain values are 12.54 × 10^−3^, 12.15 × 10^−3^, and 10.3 × 10^−3^, respectively, when the temperature reached 450 K, as shown in Fig. 7a. This implies the appearance of constraints to the epoxy chain movements is due to their interactions with SiC NWs or micron particles. Moreover, the network formed by SiC NWs can be served as framework to stabilize the molecular chains shift effectively with increasing temperature. It is believed that these decreases in thermal strain are able to reduce the coefficient thermal expansion mismatch with silicon and insulating ceramics on the basis of Hook’s law and the Poisson ratio^63^. Therefore, the coefficient of thermal expansion (CTE) curves display the same trend that can be observed in Fig. 7b. All the curves show an obviously gentle slope after the temperature exceeds 360 K. In addition, the epoxy/SiC NWs composite exhibits lower slope and maintains the slope up to 460 K, which is the end of measured temperature. Materials with lower CTE value is expected to have lower thermal strain, which will find potential application as heating sinks structural materials with improved thermal properties. Furthermore, we noticed that the CTE of our samples are comparable or much lower than other nanowires or particles reinforced polymer composites, as shown in Fig. 7c. The CTE of epoxy/SiC NWs composites at 3 wt% loading and room temperature is 30 ppm K^−1^, compared to conventional nanowires based composites, this the lowest CTE value at only 3 wt% contents of SiC NWs^60–66^. This consequence indicates that the thermal expansion is significantly restrained by the introduction of nanowires. The following relation is used to determine the volume fraction of the filler for a given weight fraction, which is represented by Equation (1).

1 $${V}_{f}=\frac{{W}_{f}}{{W}_{f}+(1-{W}_{f})\frac{{\rho }_{f}}{{\rho }_{m}}}$$

Where the V_f_, W_f_, ρ_f_ and ρ_m_ are the volume fraction of fillers, the mass fraction of fillers, the density of fillers, and the density of matrix, respectively. The mass fraction of 3 wt% can be converted to 1.1 vol%. Meanwhile, the composite incorporated with 3 wt% SiC MPs show the similar property. Besides, this outstanding performance combining with high thermal conductivity result in lower TDP, which is a prominent indicator at the engineering-scale and be defined as following Equation (2).

2 $$TDP=\frac{CTE\,({K}^{-1})}{{T}_{c}(W\,{m}^{-1}{K}^{-1})}$$

TDP is a typical indicator for indicating the temperature-induced deformation of engineering material applied electronic packaging and high precision areas^1^. The lower TDP predicts greater thermal stability, on account of the high thermal conductivity and low CTE of the epoxy/SiC NWs composite. Figure 7d exhibits that the TDP of epoxy/SiC NWs composite loading with only 1.1 vol% is lower than most available materials filled in high volume fraction of fillers. Because the high aspect ratio of the SiC NWs play an important role in determining the thermal stability^64–70^. Materials with high thermal conductive and low TDP will meet wide-ranging application in the modern electronics industry.

## Conclusions

In summary, a facile method to prepare epoxy composites has been demonstrated. The thermal conductivity of epoxy/SiC NWs composites with 3 wt% filler was 0.449 Wm^−1^ K^−1^, which is increased by 106% comparing to that of neat epoxy. It is found that the thermal stability also has a certain degree of enhancement and retains the low coefficient of thermal expansion. This composite with remarkable thermal properties is crucial in thermal management, electronic packaging, and other electrical devices applications.

## Electronic supplementary material

Supplementary Information
