# Supplementary material for: Enhanced thermal conductivity of epoxy composites filled with silicon carbide nanowires
Source: Sci Rep. 2017 Jun 1;7:2606. doi: 10.1038/s41598-017-02929-0 (PMC5453999; doi:10.1038/s41598-017-02929-0)
Supplement: Supplementary file 1 — Supplementary Information [file 41598_2017_2929_MOESM1_ESM.pdf]

SUPPLEMENTARY INFORMATION

Enhanced thermal conductivity of epoxy composites filled with silicon carbide nanowires

Dianyu Shen[1,2], Zhaolin Zhan[1]*, Zhiduo Liu[2], Yong Cao[2], Li Zhou[3], Yuanli Liu[3], Wen Dai[2], Kazuhito Nishimura[4], Chaoyang Li[5], Cheng-Te Lin[2], Nan Jiang[2]*, Jinhong Yu[2]*

[1]*Faculty of Materials Science and Engineering, Kunming University of Science and Technology, Kunming 650093, China.*

[2]*Key Laboratory of Marine Materials and Related Technologies, Zhejiang Key Laboratory of Marine Materials and Protective Technologies, Ningbo Institute of Materials Technology and Engineering, Chinese Academy of Sciences, Ningbo 315201, China.*

[3]*College of Materials Science and Engineering, Guilin University of Technology, Guilin 541004, China.*

[4]*Advanced Nano-processing Engineering Lab, Mechanical Systems Engineering, Kogakuin University, Kochi, 780-0805, Japan.*

[5]*Research Institute& School of Systems Engineering, Kochi University of Technology, Kami City, Kochi 782-8502, Japan.*

Corresponding author, Electronic mail:* ***zl_zhan@sohu.com (Z.L. Zhan); jiangnan@nimte.ac.cn (N.Jiang); yujinhong@nimte.ac.cn (J.H. Yu)*

SUPPLEMENT

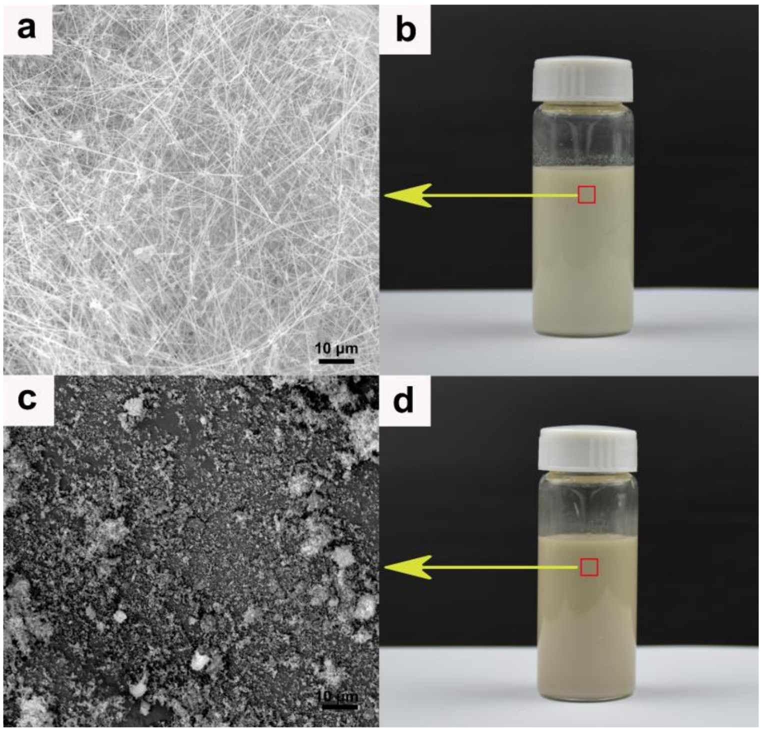

Figure S1. (a) SEM image of SiC NWs; (b) photograph of SiC NWs in ethanol; (c) SEM image of SiC MPs; (d) photograph of SiC MPs in ethanol.

Figure S1a and **c** show the SEM images of SiC NWs and SiC MPs. **Figure S1b** and **d** are the pictures of SiC NWs and SiC MPs dispersed in ethanol in an ultrasonic bath for 0.5 h. As showing in pictures the two powers can homogeneous disperse in ethanol. Meanwhile, the color of two kinds of suspension liquid is different. The SiC NWs display a milk white color. Compare to SiC NWs, the SiC MPs show a shallow brown color.

As shown in **Figure S2**, it can be noted that all the samples display similar thermal behavior and merely a one-step decomposition, indicating that the existence of the SiC NWs or SiC MPs did not significantly change the degradation mechanism of the epoxy matrix. **Figure S2a** and **b** reveal the main weight loss occur around 330-430 ℃. The chosen characteristic thermal parameters are 5% weight loss temperature ($T_{d5\%}$). As summarized in the **Table 1**, the $T_{d5\%}$of neat epoxy is 329.5 ℃. However, the $T_{d5\%}$ of epoxy composites with 0.5, 1.0, 1.5, 2.0, 2.5, and 3.0 wt% SiC NWs are 330.4, 349.1, 332.3, 343.5, 343.0, and 337.2 ℃; and the SiC MPs are 337.1, 339.5, 340.6, 346.6, 350.9, and 336.8 ℃, respectively. It is suggested that SiC NWs or SiC MPs can enhance the thermal stability of

epoxy composites. The 0.1 wt% epoxy/SiC NWs and 2.5 wt% epoxy/SiC MPs display the highest $T_{d5\%}$ compare to respective other composites. It can be also observed the char yields of all composites are increased in comparison with these of the neat epoxy. Moreover, the highest thermal degradation rate temperature, which is maximum degradation temperature (T_{max}), are shown in DTG curves in **Figure S2c** and **d**. The T_{max} of the epoxy/SiC NWs composites with 0.5, 1.0, 1.5, 2.0, 2.5, and 3.0 wt% are 374.6, 379.2, 376.4, 379.5, 379.7, and 377.3 ℃; and the epoxy/SiC MPs composites are 379.2, 376.8, 379.6, 378.8, 382.5, and 377.2 ℃, respectively. The patterns reveals that the T_{max} of the epoxy composites are also slightly improved by introduced the SiC NWs or SiC MPs compare to the T_{max} =374.5 ℃ of neat epoxy.

Table 1 Thermal properties of epoxy composites.

**SiC NWs**	**0.5 wt%**	**1.0 wt%**	**1.5 wt%**	**2.0 wt%**	**2.5 wt%**	**3.0 wt%**
**$T_{d5\%}$**	330.4	349.1	332.3	343.5	343.0	337.2
**T_{max}**	374.6	379.2	376.4	379.5	379.7	377.3
**SiC MPs**	**0.5 wt%**	**1.0 wt%**	**1.5 wt%**	**2.0 wt%**	**2.5 wt%**	**3.0 wt%**
**$T_{d5\%}$**	337.1	339.5	340.6	346.6	350.9	336.8
**T_{max}**	379.2	376.8	379.6	378.8	382.5	377.2

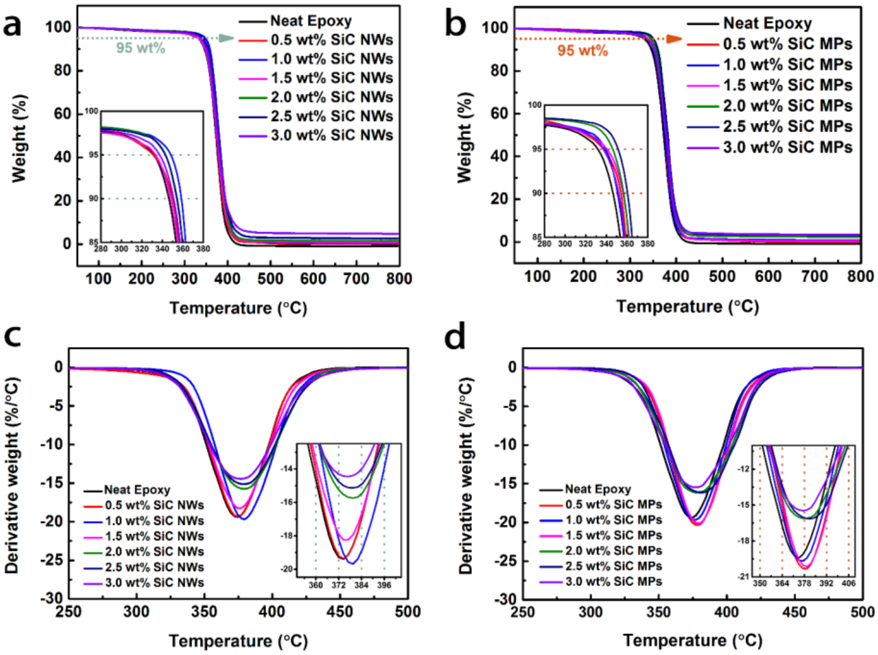

Figure S2. (a) TGA and (c) DTG curves of the neat epoxy and epoxy/SiC NWs composites, (b)TGA and (d) DTG curves of the neat epoxy and epoxy/SiC MPs composites.
